# Trends and Characteristics of New Drug Approvals in China, 2011–2021

**DOI:** 10.1007/s43441-022-00472-3

**Published:** 2022-11-02

**Authors:** Ling Su, Sen Liu, Guanqiao Li, Cuicui Xie, Huan Yang, Yang Liu, Chen Yin, Xiaoyuan Chen

**Affiliations:** 1 Yeehong Business School, Shenyang Pharmaceutical University, Shenyang, China; 2 Lilly Asia Ventures (LAV), No. 168 Hubin Road, Suite 2909, Huangpu District, Shanghai, 200021 China; 3 Tsinghua Clinical Research Institute (TCRI), School of Medicine,, Tsinghua University, Beijing, 100084 China; 4 Vanke School of Public Health, Tsinghua University, Beijing, China; 5 Pharmcube (Beijing) Co., Ltd., Beijing, China; 6 Office of Clinical Trial Institute, Beijing Tsinghua Changgung Hospital, Beijing, China

**Keywords:** New drug approval, NDA approval time, Drug lag, Regulatory reform, China

## Abstract

**Background:**

In the past decade, the Chinese drug regulatory system has undergone many changes. A major reform starting in 2015 has significantly reshaped the regulatory processes. It was important to assess the impact of the reform on new drug approvals in China.

**Method:**

We analyzed the temporal trends of regulatory characteristics of the new drugs approved by the Chinese regulatory agency from 2011 to 2021, using data collected in the Pharmcube database.

**Results:**

A total of 353 new drugs were approved, including 220 small molecule drugs, 86 biological products and 47 vaccines. The annual number of new drug approvals increased dramatically since 2017, reaching a record high of 70 in 2021. The median NDA approval time was 15.4 months in 2017-2021, the shortest in the decade, and was significantly shorter than that in the pre-reform period. The newly instituted expedited pathways such as priority review (PR) and accelerated approval for urgently needed overseas drugs (UNOD) significantly reduced new drug application (NDA) approval times compared with standard review. For imported drugs, in 2017-2021, the median time difference between the first approval in the world and the approval in China was 5 years, representing significant “drug lag”. However, the proportion of the imported drugs approved in China within 3 years of its first foreign approval has increased to 24.4% in 2017-2021.

**Conclusion:**

The regulatory reform has produced significant, positive immediate outcomes in several metrics of drug regulatory approval. China’s regulatory system will continue to evolve as there still are many areas requiring further reform and improvement.

**Supplementary Information:**

The online version contains supplementary material available at 10.1007/s43441-022-00472-3.

## Introduction

China has been the second largest single-country pharmaceutical market in the world for many years. However, over the years, several hurdles in China’s drug regulatory system and practice had significantly impeded drug development activities, new drug review, and approval in China [1]. These included the overly strict requirements for clinical trial approval, lengthy regulatory review time, lack of clearly defined sponsor-agency communication channels, and the shortage of trained reviewers, to name a few. These factors had contributed to the large backlog of new drug applications and delayed access to innovative medicines and treatments [2, 3].

In August, 2015, the State Council of China issued a policy document entitled “Opinions on the Reform of Review and Approval Process for Drugs and Medical Devices,” marking the beginning of the regulatory reform through the next several years [4–6]. Implementation of a series of reform policies has led to the revision of the Drug Administration Law, the adoption of the new Vaccine Administration Law, and the re-write of many important regulations. All of these have fundamentally reshaped the regulatory environment in China.

In June 2017, the Assembly of the International Council for Harmonisation of Technical Requirements for Pharmaceuticals for Human Use (ICH) approved the then Chinese regulatory agency, China Food and Drug Administration (CFDA, predecessor of today’s National Medical Products Administration, NMPA) as a Regulatory Member of ICH [7]. China’s joining ICH was an important milestone in its regulatory history. It signified that the agency was prepared to adopt ICH technical requirements for drug registration and to become a global player in drug approval and regulation [8].

It has been more than six years since the landmark reform started. However, there is a lack of reports examining the impact of the reform measures on the metrics of drug review and approval across therapeutic areas and modalities. Therefore, we set out to investigate quantitatively the trend and characteristics of regulatory review and approval of new drugs in 2011–2021, comparing the results in 2017–2021 with those in prior years. The speed with which a drug regulatory agency evaluates and approves new drugs is an important indicator of regulatory capability and efficiency. In this research, we analyzed the temporal trends of the number of new drugs approved and the approval times by the Chinese regulatory agency. We also examined how other characteristics such as regulatory programs, oncology drugs, rare disease drug status, and number of the review cycles may have influenced the trends. At the same time, Chinese domestic pharma and biotech companies have evolved rapidly over the past few years. They develop new drugs through in-license or in-house discoveries [9]. Since the Chinese regulatory system has historically instituted different drug application processes for domestic (locally manufactured) drugs and for imported drugs, it was also of interest to analyze the data comparing domestic drug approvals with import drug approvals.

## Methods

We defined a “new drug” as a new chemical entity (small molecule drug, excluding new salt, new ester, or new combination), a novel biological product or a novel vaccine, excluding traditional Chinese medicines, blood products, and biosimilars, that received marketing authorization approval for the first time in mainland China. All new drugs approved between January 1, 2011, and December 31, 2021, were included. All data were collected from the Pharmcube database, a proprietary platform of drug information in China curated from the official databases of China’s National Medical Products Administration (NMPA), Center for Drug Evaluation (CDE), and other public sources. Basic and regulatory characteristics were extracted and manually verified, including therapeutic class, dates of receipt of new drug application (NDA) and date of the first approval in China, number of review cycles, qualification of expedited programs such as priority review, and manufacturing site of the drug [manufactured in China (domestic) or outside China (imported)]. When a drug has multiple approved indications, the approval date of the first indication was used.

The key parameter of interest, NDA approval time was defined as the number of months from the receipt of the marketing authorization application to the approval. Numerical data were presented with median and interquartile ranges. A nonparametric Kruskal–Wallis H test was performed to examine the differences in NDA approval times between different time periods and between other characteristics. Statistical analyses were performed using IBM SPSS Statistics version 21 and GraphPad Prism version 8.0. A two-tailed *p* value < 0.05 was considered statistically significant.

## Results

**Fig. 1:**
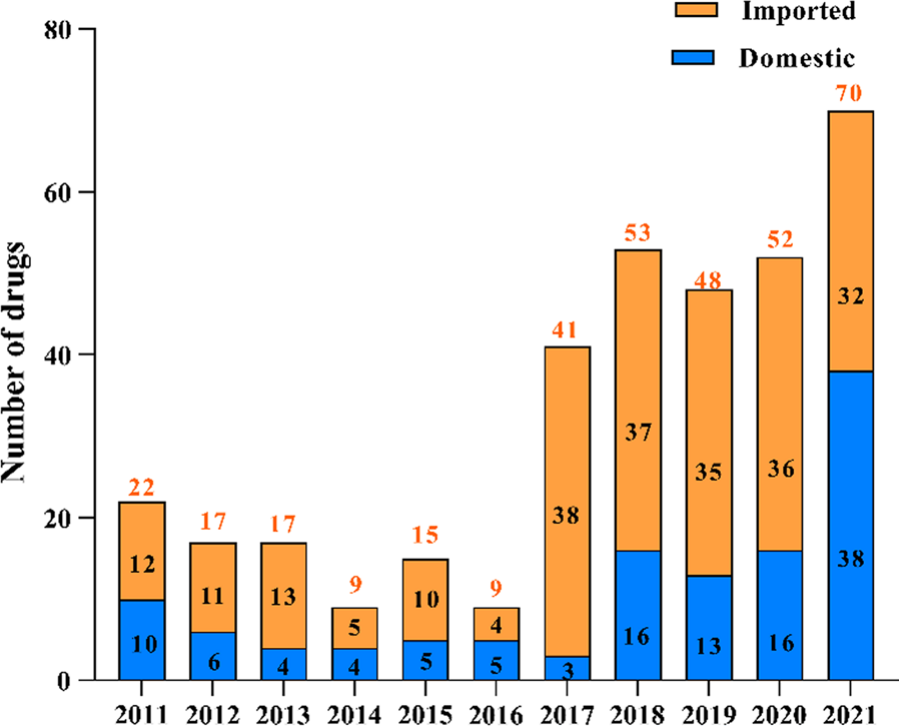
Number of new drugs approved in China, 2011–2021

In 2011–2021, a total of 353 new drugs were approved in China, including 220 small molecule drugs, 86 biological products, and 47 vaccines. Of the approvals, 233 (66%) were imported drugs and 120 (34%) were domestic drugs. The top 5 therapeutic classes of the new drugs were oncology (94, 27%), anti-infections (53, 15%), prophylactic vaccines (47, 13%), endocrinology and metabolism (37, 11%), and cardiovascular diseases (24, 7%) (Supplemental Table 1). The number of new drugs approved remained relatively steady at around 9–22 per year in 2011–2015. The year of 2016 recorded the lowest number of new drugs approved, followed by a surge starting 2017. In 2021, 70 new drugs were approved, the largest number of approvals in the period (Fig. 1).

**Fig. 2:**
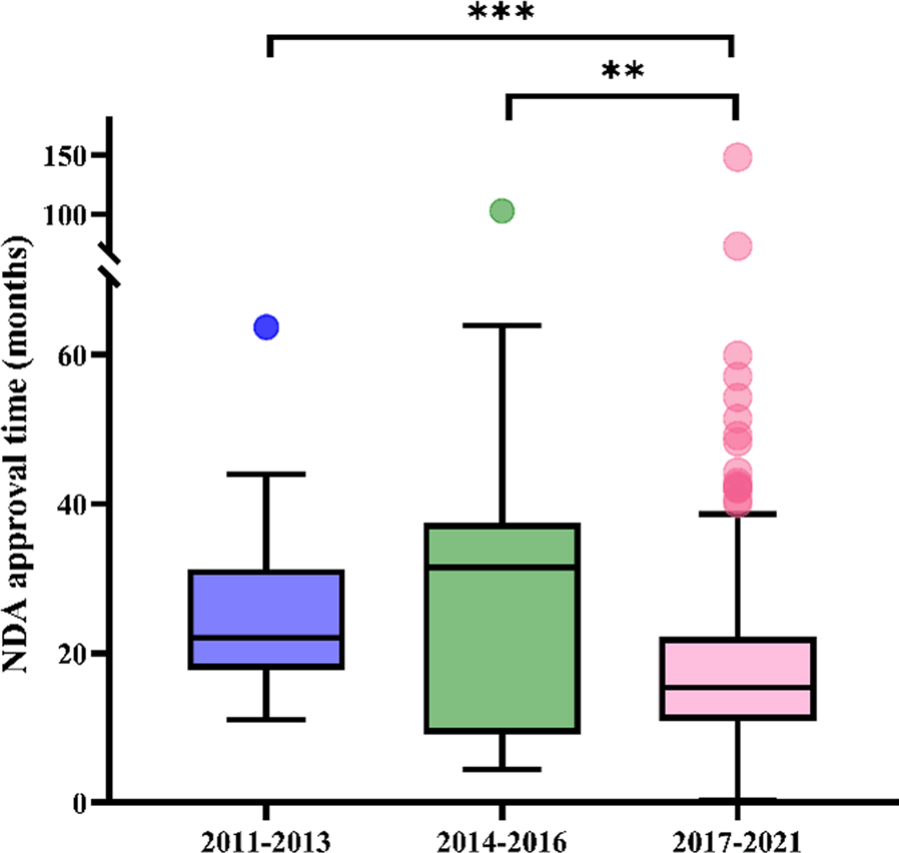
NDA approval times for new drugs approved in China, 2011–2021, defined as the time from the receipt of new drug application to approval of the first indication. Box plots indicate interquartile ranges in shaded areas and maximum and minimum values in whiskers, and the dots indicate outliers. **p < 0.01; ***p < 0.001

To investigate the characteristics of new drug approvals over time, we analyzed the data according to three time periods based on the year of approval: 2011–2013, 2014–2016, and 2017–2021. Three approvals were excluded from the analysis due to missing data on the NDA receipt date. Also excluded were four COVID-19 vaccines and two anti-SARS-CoV-2 antibodies that were approved in 2021 through the Special Approval Procedure for Public Health Emergency. This special procedure does not follow the routine review and approval process and timelines. As such, including these approvals would have biased the analysis and comparison of approval times. As a result, 344 drug approvals were included in the analyses of NDA approval times. Overall, the median NDA approval time of all drugs approved (small molecules, biologics, and vaccines) was the shortest in 2017–2021 (median, 15.4 months; IQR: 11.3–22.2 months). It was statistically significantly shorter than those in 2011–2013 (median, 22.1 months; IQR: 18.2–31.2 months; *p* < 0.001) and in 2014–2016 (median, 31.5 months; IQR: 9.6–36.4 months; *p* < 0.01) (Fig. 2 and Supplemental Table 2). This was consistent across the three therapeutic modalities. The median NDA approval times in 2017–2021 for small molecule drugs, biological products, and vaccines were 15.7, 14.2, and 22.0 months, respectively. All were shorter than those in prior periods. When comparing with the period of 2011–2013, the differences were statistically significant for small molecule drugs (15.7 vs 20.4 months, *p* < 0.05) and for biological products (14.2 vs 21.3 months, *p* < 0.05). For vaccines, the difference was statistically significant when comparing with 2014–2016 (22.0 vs 33.6 months, *p* < 0.05).

**Fig. 3:**
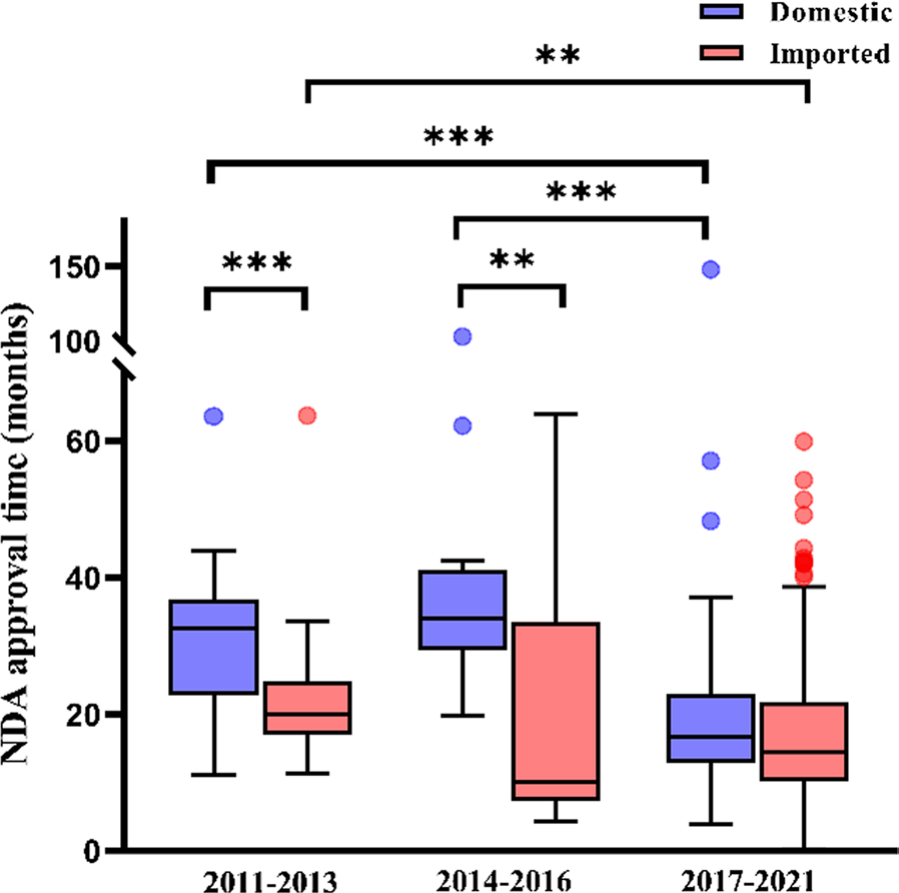
NDA approval times for domestic and imported new drugs approved in China, 2011–2021, defined as the time from the receipt of new drug application to approval of the first indication. Box plots indicate interquartile ranges in shaded areas and maximum and minimum values in whiskers, and the dots indicate outliers. **p < 0.01; ***, *p* < 0.001

It was of interest to assess whether the NDA approval times differed between imported and domestic drugs. As shown in Fig. 3 and Supplemental Table 3, in all three time periods, the median NDA approval times were shorter for imported drugs than domestic drugs. The differences were statistically significant in 2011–2013 (19.9 vs 32.5 months, *p* < 0.001) and 2014–2016 (10.0 vs 34.1 months, *p* < 0.01) and were no longer statistically significant in 2017–2021 (14.5 vs 16.7 months, *p* > 0.05). While consistent with the overall trend that the NDA approval times for both domestic and imported drugs have decreased in recent years, the reduction was much greater for domestic drugs. The gap between imported and domestic drugs in NDA approval times was only 2.2 months in 2017–2021.

We examined if more review cycles were associated with increased NDA approval time. Overall, we found that two or more review cycles significantly increased the approval times (11.7 months, one-cycle review vs 20.6 months, two or more cycle reviews, *p* < 0.001). The trend was consistent across the three time periods, but the difference in NDA approval time became smaller in the most recent period of 2017–2021(Supplemental Table 4).

**Fig. 4:**
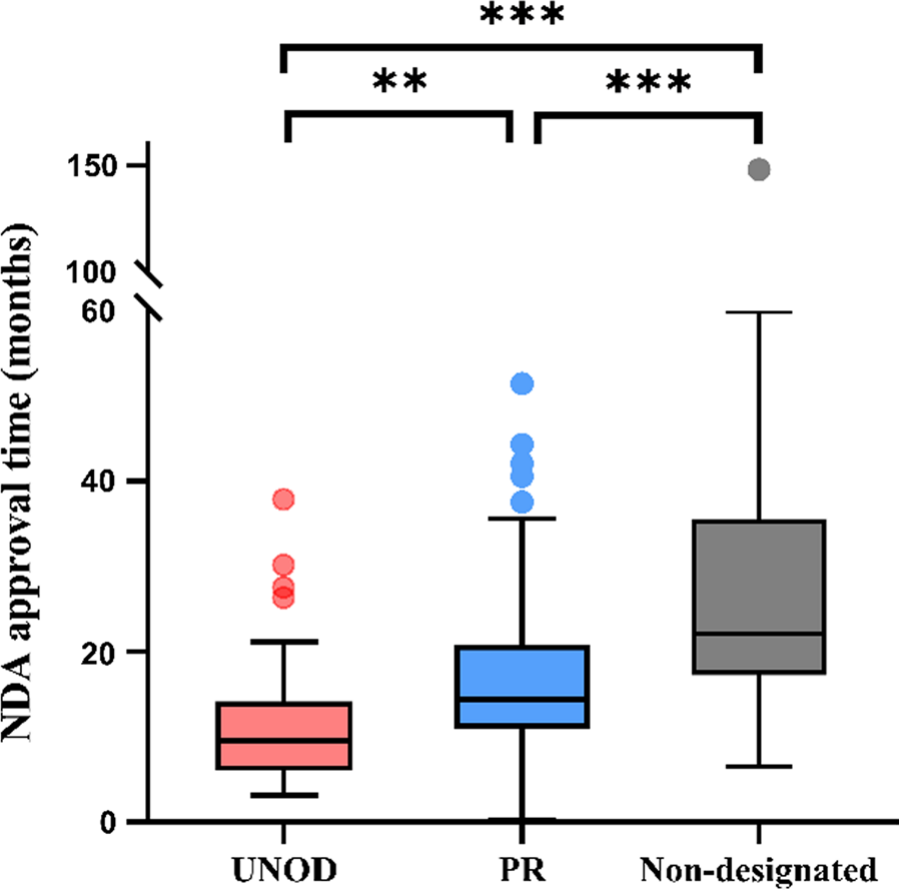
NDA approval times for new drugs approved with special designations in China from 2017 (including 2017), defined as the time from receipt of the new drug application to approval of the first indication. UNOD: urgently needed overseas drugs; PR: priority review. Box plots indicate interquartile ranges in shaded areas and maximum and minimum values in whiskers, and the dots indicate outliers. **p < 0.01; ***p < 0.001

As part of the regulatory reform, the Chinese regulatory agency introduced, among other measures, priority review (PR) in 2016 and accelerated approval for foreign-approved urgently needed overseas drugs (UNOD) in 2018. To investigate the impact of such designations on NDA approval time, we analyzed the data for the period 2017–2021. Of 264 new drugs approved in this period, excluding one approval with missing NDA receipt date, four COVID-19 vaccines and two anti-SARS-CoV-2 antibodies, 257 were included in the analysis (Supplemental Table 5). There were 156 PRs, 25 UNODs, 9 PR/UNOD dual designations, and 67 non-designated. For the simplicity of analysis, the PR/UNOD dual designations were included in the UNOD group in analysis. In the PR group, oncology drugs accounted for a large proportion of the applications (42%), followed by anti-infectious drugs (16%) and neurologic drugs (8%). The results showed that, compared to standard approvals (22.1 months), both PR and UNOD significantly decreased the median NDA approval times (14.4 months, *p* < 0.001, and 9.6 months, *p* < 0.001, respectively). Furthermore, the median NDA approval time for UNOD was also statistically shorter than that of PR (*p* < 0.01) (Fig. 4, Supplemental Table 5).

**Fig. 5:**
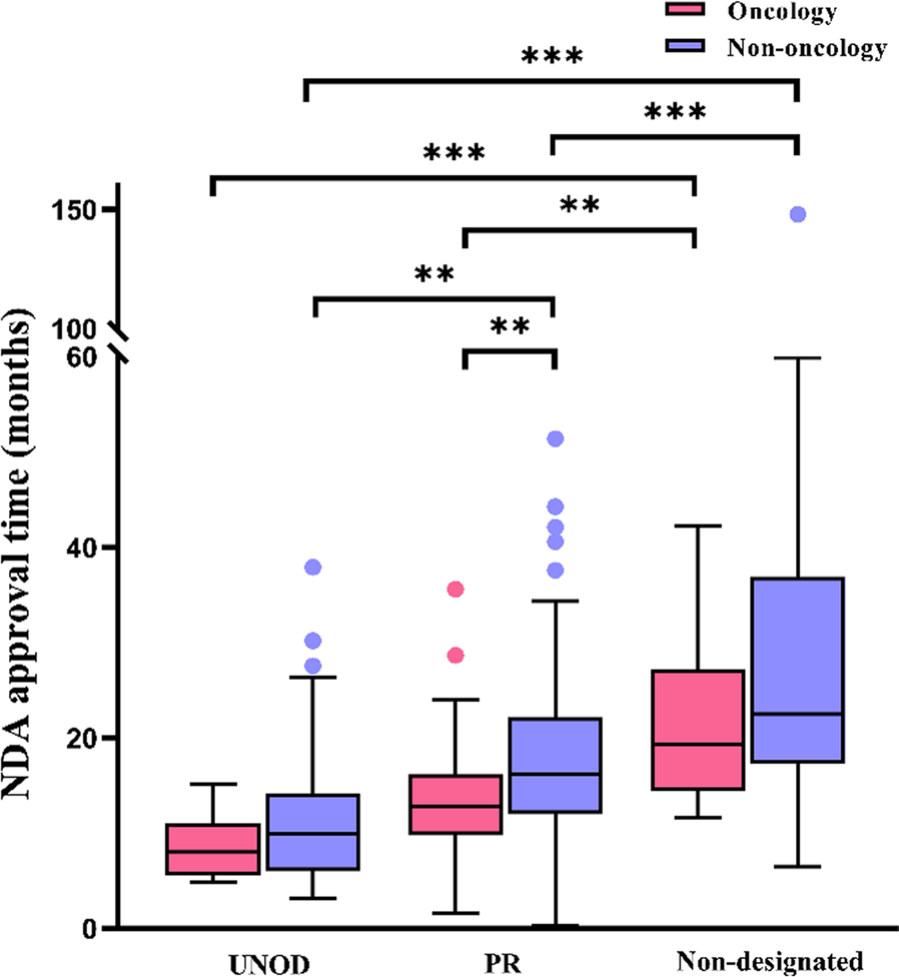
NDA approval times for new oncology and non-oncology drugs approved with priority review (PR) or urgently needed overseas drugs (UNOD) designation in China from 2017 (including 2017), defined as the time from the receipt of new drug application to approval of the first indication. Box plots indicate interquartile ranges in shaded areas and maximum and minimum values in whiskers, and the dots indicate outliers. **p < 0.01; ***p < 0.001

In light of the high publicity and attention toward oncology drugs in recent years, we investigated if oncology drugs per se carried an advantage in terms of NDA approval time. As shown in Fig. 5 and Supplemental Table 6, oncology drugs were associated with shorter NDA approval time. Within the PR category, NDA approval time for oncology drugs was statistically significantly shorter that for non-oncology drugs (12.8 months vs 16.2 months; *p* < 0.01). Compared with non-oncology drugs, oncology drugs were also associated with shorter NDA approval times within the categories of UNOD (8.1 months, oncology drugs vs 9.9 months, non-oncology drugs) and standard approval (19.4 months, oncology drugs vs 22.5 months, non-oncology drugs), but the differences were not statistically significant.

In May 2018, several Chinese government agencies jointly published the first list of rare diseases, officially recognizing rare disease status in China. There were 21 drugs for rare disease approved in 2018–2021. In this period immediately following the issuance of the list, our analysis showed that the rare disease drug status was not associated with shorter NDA approval time (14.2 months, rare disease drugs vs 15.2 months, non-rare disease drugs, *p* > 0.05) (Supplemental Table 7).

For imported drugs, we investigated the trend of “drug lag,” defined as the time difference between a drug’s first approval in any other country in the world and its approval in China. Among 233 imported drugs, 228 drugs (Table 1) with complete data on the first foreign approval were included in the analysis. Based on the year of China’s approval, we found that the median lag times were 5.1 years, 7.2 years, and 5.0 years for imported drugs approved in 2011–2013, 2014–2016, and 2017–2021, respectively. There was a sign of reduction of the lag time in the most recent period, but the difference was not statistically significant. The proportion of drugs approved in China within 3 years of its first approval increased from 11.1% in 2014–2016, the lowest in the past decade, to 24.4% in 2017–2021, but it still was 2.1% less than that in 2011–2013 (26.5%). The proportion of drug approvals with a drug lag of 5 years or more has decreased to 51.1% in 2017–2021, from 58.8 to 66.7% in 2011–2013 and 2014–2016, respectively (Table 1).

**Table 1:** Approval lag of imported drugs in different time periods

Year of approval in China	2011–2013	2014–2016	2017–2021
Number of imported drugs approved^*^	34	18	176
Lag time (year), median (IQR)	5.1 (2.7–8.5)	7.2 (4.2–10.0)	5.0 (3.1–9.2)
Number and proportion of imported drugs approved with various drug lags			
0–3 years	9 (26.5%)	2 (11.1%)	43 (24.4%)
3–5 years	5 (14.7%)	4 (22.2%)	43 (24.4%)
> 5 years	20 (58.8%)	12 (66.7%)	90 (51.1%)

^*^228 imported drugs with complete data were included in the analysis

## Discussion

The reform that started in 2015 has brought about an overhaul to the Chinese regulatory system. The initial focus of the reform was to reduce the massive backlog of drug clinical trial and marketing applications, which stood at its peak of 22,000 in September 2015, and to improve the efficiency of the regulatory review process. A series of measures were implemented. For examples, a mandatory self-examination and inspection program of clinical trial data for 1622 applications was carried out in 2015 to ensure data authenticity and integrity in regulatory filing and to crack down on potential data fraud. Other key measures included a new set of criteria for priority review, an updated requirement of filing a notification instead of obtaining approval for bioequivalent studies of chemical drugs, an enhanced sponsor-reviewer communication mechanism, as well as streamlined internal working procedures and expansion of reviewing staff in the Center for Drug Evaluation (CDE). These measures had helped eliminate the backlog of applications by the end of 2017. The outstanding number of applications has since remained stable in the proximity of 4500.

Starting in late 2016, the scope of the reform was broadened, deepening to the fundamental regulatory processes. The goals were shifted to become focused on comprehensively restructuring the regulatory system and encouraging innovation. A broad range of policy proposals was introduced, many of which were codified in the revision of the Drug Administration Law in December, 2019, or included in the subsequent re-write of key regulations, such as Drug Registration Regulation, Drug Manufacturing Regulation, Good Clinical Practice, and other regulatory directives. Many internal procedures were also updated or created to adapt to the mandates of the newly revised laws and regulations. China’s joining ICH also facilitated the adoption of ICH technical guidelines. As such, the new regulatory framework was largely established and the regulatory system entered a “norming” stage.

The number of new drugs approved each year and the regulatory approval time provide suitable metrics to assess the overall performance of a regulatory review system, and in our research, the overall impact of the reform. Our analysis showed that there clearly was a surge in 2017 with 41 new drugs approved compared with only nine new drug approvals in the year before (Fig. 1). In subsequent years, the number of new drugs approved each year continued to remain at a high level, reaching a record high of 70 in 2021. The COVID-19 pandemic in 2020 and 2021 did not appear to have hindered the new drug review and approval activities in China. It is not surprising that overall, imported drugs accounted for approximately two-thirds of the new drugs approved in the period studied, because the domestic pharmaceutical industry has historically not been a source of innovative drugs for China. On the other hand, the number of approved new drugs from domestic companies has been increasing in recent years. The local biotech and pharma industry is rapidly growing and evolving, and has started to discover and develop new drugs, albeit very few first-in-class molecules, for Chinese and global markets. It is notable that the number of domestic drug approvals had surpassed imported drugs in 2021 (38 vs. 32) (Fig. 1). While it remains to be seen if such a high proportion of domestic new drug approvals will sustain, we anticipate that in future years, a substantial percentage of new drug approvals will be from local Chinese biopharma companies. This reflects the favorable regulatory policies supporting a growing local innovation ecosystem and the rapid rise of China home-grown products in global R&D pipeline [10].

In the analyses of NDA approval times, we analyzed the data according to three time periods based on the year of approval: 2011–2013, 2014–2016, and 2017–2021. The last period would represent the immediate outcome resulting from the recent regulatory reform. The choice of defining the other two periods was based on the following considerations. Firstly, in 2011, amid increasing number of applications, the CDE underwent a re-structuring of review offices to better coordinate review tasks given the scarce internal review resources available at that time. Secondly, several more restrictive regulatory practices were implemented in 2014, adding additional administrative steps in the approval process. For example, for imported drugs, an additional step of application and approval for a registration trial was required even if a clinical study was conducted in China as part of the multi-regional clinical trial (MRCT) program. It was widely considered as purely bureaucratic without adding any value. The practice was scrapped in October, 2017, but it did prolong NDA approval times, as we have shown in the study. Therefore, the comparisons across these three periods would help assess the impact of the reform (2017–2021) in comparison with the “old normal” (2011–2013) and to the “low point” (2014–2016) over the last decade.

Before the regulatory reform, it took about 2 years or more for an NDA to get approved in China. However, the NDA approval time in 2017–2021 had decreased and was statistically significantly shorter than that in each of the two prior periods. The median NDA approval time of 15.4 months in 2017–2021 (Supplemental Table 2) represents a reduction of 51% and 30% from 2014–2016 and 2011–2013, respectively. When data were analyzed separately for different therapeutic modalities, namely, small molecule drugs, biologic products, and vaccines, similar trends were observed. These findings provided direct evidence of the initial positive impact of the reform. A recent report focusing on the innovative drug development in China also showed a significant reduction in NDA approval for innovative drugs in the post-reform period [11]. The updated Drug Registration Regulation stipulates a statutory NDA review and approval time limit of 225 working days (approximately 10.3 months) for standard review and 155 working days (approximately 7.1 months) for priority review, which are getting closer to the US FDA’s benchmark. We anticipate that the NDA approval time in China will further decrease as more applications are now covered under this approval time limit mandate.

Historically, the Chinese regulatory system has different review approaches for “imported drugs,” i.e., drugs manufactured outside mainland China, and locally manufactured drugs. They were subject to different administrative procedures and requirements. For example, for imported drugs, the drug must at least be in Phase II trial stage before a clinical trial can be conducted in China. Furthermore, to file an NDA in China, the imported drug would typically have already been approved in another country. Such restrictions were lifted in October, 2017 as part of the reform, and the difference between imported drugs and locally manufactured drugs has become much less evident. There was a perception that the regulatory practice might favor domestic new drugs. Our finding indicated the contrary and provided interesting insight. We found that in 2011–2013 and 2014–2016, imported drugs had a significantly shorter NDA approval time than domestic new drugs (Supplemental Table 3). In 2017–2021, the NDA approval time was still slightly longer for domestic new drugs, but the difference was only 2.2 months and was no longer statistically significant. A probable explanation is that for domestic new drugs, pre-approval on-site inspection of the manufacturing site was a requirement and was performed typically after the NDA technical review. This sequential procedure added significant amount of time to the overall process. On the other hand, for imported drugs, such inspection was not mandatory and was rarely conducted in practice. This procedural disparity may have led to a time difference favoring imported drug applications. With the implementation of the updated Drug Registration Regulation in July, 2020, pre-approval inspection is now conducted in parallel with the technical review. Our finding suggests that this procedural change may have contributed to the shortened approval time.

Expedited regulatory programs such as priority review, conditional approval, and breakthrough designations, are important mechanisms to bring the drugs for serious and life-threatening diseases to the patients as fast as possible [12, 13]. The Chinese CDE first introduced priority review (PR) in 2016 as a mechanism to prioritize review of qualified applications to reduce the application backlog. It was later further refined and became one of the formal expedited programs in 2020, along with conditional approval, breakthrough designation, and special approval for public health emergency [14]. The current requirements and features of these programs closely resemble the US FDA’s expedited programs. A key feature of PR is the shortened NDA review and approval time of 155 working days. The UNOD program is a unique, time-limited policy instituted by the Chinese agency in November, 2018 [15]. It designates certain drugs approved in the USA, the European Union, and Japan in the past 10 years that are considered urgent medicines needed for Chinese patients. These drugs are qualified for speedy approval, mainly based on clinical trial data generated in other countries and with a technical review clock of 6 months, or 3 months for rare disease drugs. Between November, 2018 and November, 2020, the CDE designated 81 drugs in this category. Up to the end of 2021, 51 of these drugs had been approved [16]. In our analysis, as expected, both PR and UNOD designations conferred significantly shorter NDA approval times as compared with standard reviews. The NDA approval time associated with UNOD was further reduced and was statistically shorter than that of PR (Fig. 4). This finding confirmed the significant advantage in approval time reduction that PR and UNOD were designed for.

Cancer is a devastating disease affecting millions and thousands of people. Timely approval of new oncology drugs saves lives and has attracted a lot of public attention. Drug regulatory agencies strive to approve novel oncology drugs in an expeditious way [17]. We found that oncology drugs were associated with shorter NDA approval time than non-oncology drugs, regardless of whether or not the drug was designated as PR, UNOD, or standard review. Within the PR group, the approval time difference was statistically significant. Rendering significant clinical benefit by the new drug under evaluation is one of the main key considerations in granting priority review. In November, 2021, the CDE issued the “Guideline on Clinical Value Oriented Oncology Drug Development” [18]. This was the first regulatory guideline in China focusing on patient-centric drug development. Our finding also reflects the strong drive to accelerate the review and to improve accessibility of new oncology drugs to cancer patients.

Our analysis did not find an association between the rare disease drug status and a shorter NDA approval time in 2018–2021. However, this finding should be interpreted with caution. Firstly, the NDAs of some of the rare disease drugs approved in this period were likely submitted before May, 2018 when the list of rare diseases was issued. The NDA time analysis may be confounded. Secondly, according to regulations, rare disease drug status in itself is not a sufficient condition for PR which confers a shortened NDA time. The effect on NDA approval time of rare disease drugs is likely mediated through other attributes of the drug, such as innovative new drug (category 1 drug) or innovative improved new drug (category 2 drug), or through expedited programs or mechanisms, such as PR, conditional approval, breakthrough therapy designation, or UNOD, for which the drug may be qualified.

Drug lag is often used as a measure of how far behind patients in one country have access to the newest or the most advanced therapies compared with those in other countries [19, 20]. Our findings suggest that in recent years, the proportion of new drugs approved in China with shorter drug lags has increased and the proportion of those with longer drug lags has decreased. The drug lag may be reduced if the trend continues. Nevertheless, the lag of 5 years in the most recent period studied is still quite striking. Drug lag is a long overdue issue in China [3]. As has been shown, the NDA approval process per se was not a contributing factor causing the delay as imported drugs had a shorter NDA approval time. Other factors may have played a major role. Local clinical trial data are typically needed in most cases to support a new drug marketing authorization application in China. In the past, the lengthy approval time for clinical trials had often precluded China’s joining global development trials. As a result, one or more local clinical trials were often conducted to generate Chinese data following global trials or even after foreign approval. As the reform progressed, the measures tackling regulatory hurdles for clinical trials and global clinical data for registration were implemented. In 2018, the clinical trial approval process was streamlined with a 60-working days approval clock, and a regulatory guideline of accepting foreign clinical data was issued by the NMPA. Furthermore, the ICH E17 guideline was officially adopted in China in November, 2019. More multinational pharmaceutical companies have included China in global clinical trials. But the extent to which global clinical trials have supported imported drug registration in China is currently unclear. There are many legacy drug development programs ongoing and NDAs in process. Substantial time and efforts are still needed to adequately address the drug lag in China. The new regulatory framework in China and China’s being a member of the ICH offers great opportunities for simultaneous drug development and regulatory convergence. A paradigm shift is needed for both the regulatory reviewing body and the industry to truly embrace the notion of global drug development. Science-based assessment of potential ethnic or regional difference, along with the evaluation of new drug’s safety and efficacy based on global clinical data, will help obviate the need for local clinical trials. Multinational pharmaceutical industry will benefit from the strategy of including China very early in the global clinical development footprint to enable late-stage multi-regional clinical trials to shorten overall clinical development time and to file marketing authorization applications simultaneously in multiple regions including China.

Our study has some limitations. Firstly, in calculating NDA approval times, due to data availability, we were not able to ascertain the time spent by the applicant to prepare and submit a detailed dossier and supplemental information as requested by the CDE. That information would have helped obtain more precise estimates of the actual duration that the CDE used to perform the technical review. Nevertheless, our objective was to assess the impact of the reform by comparing the approval times across different time periods, and we do not expect it would bring excessive bias to our analyses and the interpretation of results. Secondly, we have divided the decade into three time periods for analysis based on certain events in China’s regulatory history. Giving the rapid evolution and progress of the regulatory reform every year, grouping the years in the reform era into one group may have underestimated the true impact of the reform measures, particularly for the most recent one or two years.

## Conclusion and Outlook

The unprecedent regulatory reform in China has made a game-changing impact on China’s drug regulatory administration system and has produced encouraging outcomes in several key metrics. The annual number of new drug approvals has steadily increased and reached a record high in 2021. The NDA approval time was significantly shortened compared with the pre-reform period. The newly instituted expedited regulatory pathways are taking effect. More imported drugs are entering China sooner, suggesting a positive prospect of reduction of drug lag. Therefore, patient’s accessibility to innovative and the advanced treatments is being improved. Moving forward, China’s regulatory system will continue to evolve as there still are many areas requiring further reform and improvement. Transforming such a complex system in an ever-changing scientific, economic, and political context is a daunting task and the course of the reform will not be uneventful. However, policy reversal or major disruption is not expected. It is therefore imperative that all stakeholders collaborate closely to realize the benefits of the reform and to support NMPA to become a Stringent Regulatory Agency with high efficiency and quality regulatory decision-making.

## Supplementary Information

Below is the link to the electronic supplementary material.

Supplementary file1 (PDF 156 kb)

## References

[1] Bajaj G, Gupta M, Wang HH (2019). Challenges and opportunities with oncology drug development in China. Clin Pharmacol Ther.

[2] Shao L, Xu L, Li Q (2016). Regulatory watch: innovative drug availability in China. Nat Rev Drug Discov.

[3] Li X, Yang Y (2021). The drug lag issue: a 20-year review of China. Invest New Drugs.

[4] Zhou Q, Chen XY, Yang ZM (2017). The changing landscape of clinical trial approval processes in China. Nat Rev Clin Oncol.

[5] Xu L, Gao H, Kaitin KI (2018). Reforming China’s regulatory system. Nat Rev Drug Discov.

[6] Chen J, Zhao N (2018). Recent advances in drug development and regulatory science in China. Ther Innov Regul Sci.

[7] ICH. Press release ICH Assembly meeting in Montreal, Canada, May/June 2017. 19 June 2017. https://ich.org/news/press-release-ich-assembly-meeting-montreal-canada-mayjune-2017

[8] Yuan L, Zhang GT, Sun L (2018). The whole story of China’s joining ICH and its significance. China Food Drug Adm Mag.

[9] Mullard A (2017). Chinese biopharma starts feeding the global pipeline. Nat Rev Drug Discov.

[10] IQVIA Institute for Human Data Science. Global Trends in R&D: Overview Through 2021. https://www.iqvia.com/-/media/iqvia/pdfs/institute-reports/global-trends-in-r-and-d-2022/iqvia-institute-global-trends-in-randd-to-2021.pdf

[11] Su X, Wang H, Zhao N (2021). Trends in innovative drug development in China. Nat Rev Drug Discov.

[12] Cox EM, Edmund AV, Kratz E (2020). Regulatory affairs 101: Introduction to expedited regulatory pathways. Clin Transl Sci.

[13] Richardson E, Daniel G, Joy DR (2018). Regional approaches to expedited drug development and review: can regulatory harmonization improve outcomes?. Ther Innov Regul Sci.

[14] Li G, Liu Y, Xie C (2021). Characteristics of expedited programmes for cancer drug approval in China. Nat Rev Drug Discov.

[15] National Medical Products Administration and National Health Commission. Working Procedure of Review and Approval of Clinically Urgent Needed Overseas New Drugs. https://www.nmpa.gov.cn/xxgk/ggtg/qtggtg/20181030171201646.html

[16] Center for Drug Evaluation, National Medical Products Administration. The 2021 Drug Review Annual Report. https://www.cde.org.cn/main/news/viewInfoCommon/f92b7bdf775bbf4c4dc3a762f343cdc8

[17] Wang S, Yang Q, Deng L (2022). An overview of cancer drugs approved through expedited approval programs and orphan medicine designation globally between 2011 and 2020. Drug Discov Today.

[18] Center for Drug Evaluation, National Medical Products Administration. Guideline on Clinical Value Oriented Oncology Drug Development. https://www.cde.org.cn/main/news/viewInfoCommon/ef7bfde96c769308ad080bb7ab2f538e

[19] Mototsugu T, Mayumi I, Hiroshi S (2021). Evolving landscape of new drug approval in Japan and lags from international birth dates: retrospective regulatory analysis. Clin Pharmacol Ther.

[20] Cho I, Han E (2022). Drug lag and associated factors for approved drugs in Korea compared with the United States. Int J Environ Res Public Health.

