# Supplementary material for: Trends and Characteristics of New Drug Approvals in China, 2011–2021
Source: Ther Innov Regul Sci. 2022 Nov 2;57(2):343–51. doi: 10.1007/s43441-022-00472-3 (PMC9628473; doi:10.1007/s43441-022-00472-3)
Supplement: Supplementary file 1 — Supplementary file1 (PDF 156 kb) [file 43441_2022_472_MOESM1_ESM.pdf]

Supplemental Table 1. Distribution of new drugs approved in 2011-2021, by therapeutic class

Therapeutic Class	Count (%*)
Oncology	94 (26.6)
Anti-infections	53 (15.0)
Prophylactic vaccines	47 (13.3)
Endocrinology and metabolism	37 (10.5)
Cardiovascular	24 (6.8)
Neurology	20 (5.7)
Immunology and skeletal	16 (4.5)
Blood	13 (3.7)
Respiratory and allergy	9 (2.5)
Antipsychotics	9 (2.5)
Skin	9 (2.5)
Ophthalmic	6 (1.7)
Genitourinary	6 (1.7)
Gastrointestinal	5 (1.4)
Others	5 (1.4)
Total	353 (99.8)

*Percentage did not add up to 100 due to rounding.

Supplemental Table 2. Comparisons of NDA approval times in various time periods, by therapeutic modality and by manufacturing location (within or outside mainland China)

Category of drugs	Count	NDA approval time (month) Median (IQR)			Comparison *p*-value		
		2011-2013	2014-2016	2017-2021	2017-2021 vs. 2011-2013	2017-2021 vs. 2014-2016	2014-2016 vs. 2011-2013
All[*]	344	22.1 (18.2-31.2)	31.5 (9.6-36.4)	15.4 (11.3-22.2)	<0.001	<0.01	>0.05
Small molecule drugs	219	20.4 (16.9-26.9)	22.9 (8.6-35.2)	15.7 (11.3-22.2)	<0.05	>0.05	>0.05
Biological products	84	21.3 (20.0-26.9)	33.0 (19.8-35.7)	14.2 (10.5-19.2)	<0.05	>0.05	>0.05
Vaccines	41	34.4 (24.9-36.6)	33.6 (31.5-44.4)	22.0 (10.1-28.4)	>0.05	<0.05	>0.05
Imported drugs	231	19.9 (17.2-24.9)	10.0 (7.7-32.5)	14.5 (10.2-21.5)	<0.01	>0.05	>0.05
Domestic drugs	113	32.5 (23.1-36.6)	34.1 (31.9-40.0)	16.7 (12.8-22.4)	<0.001	<0.001	>0.05

[*]344 drug approvals were included in the analyses due to exclusions (see text for details).

Supplemental Table 3. Comparisons of NDA approval times between imported and domestic drugs, by time period

Time period	Imported drugs		Domestic drugs		*p*-value
	Count	NDA approval time (month) Median (IQR)	Count	NDA approval time (month) Median (IQR)	
2011-2013	36	19.9 (17.2-24.9)	19	32.5 (23.1-36.6)	<0.001
2014-2016	18	10.0 (7.7-32.5)	14	34.1 (31.9-40.0)	<0.01
2017-2021	177	14.5 (10.2-21.5)	80	16.7 (12.8-22.4)	>0.05

Supplemental Table 4. NDA approval times, by number of review cycles

Number of review cycles	Count	NDA approval time (month) Median (IQR)				Comparison *p*-value
		2011-2021	2011-2013	2014-2016	2017-2021	2011-2021
1	96	11.7 (6.9-17.3)	17.2 (14.4-18.9)	8.6 (6.8-9.4)	11.6 (6.3-16.1)	<0.001
⩾2	248	20.6 (13.2-30.1)	26.8 (21.6-34.1)	33.6 (31.5-40.7)	17.5 (12.5-24.7)	

Supplemental Table 5. NDA approval times, by regulatory program*

Regulatory Program	Count	NDA approval time (month) Median (IQR)	Comparison *p*-value	
Urgently needed overseas drugs (UNOD)**	34	9.6 (6.0-14.0)	UNOD vs. ND	<0.001
Priority review (PR)	156	14.4 (11.1-20.7)	PR vs. ND	<0.001
Non-designated (ND)	67	22.1 (17.4-35.2)	UNOD vs. PR	<0.01

* 257 drug approvals in time period of 2017-2021 were included due to exclusions (see text for details).

** Including 9 PR/UNOD dual designations.

Supplemental Table 6. Comparisons of NDA approval times between oncology and non-oncology drugs, by regulatory program

Regulatory Program	Oncology drugs		Non-oncology drugs		*p*-value
	Count	NDA approval time (month) Median (IQR)	Count	NDA approval time (month) Median (IQR)	
Urgently needed overseas drugs (UNOD)*	6	8.1 (6.1-9.6)	28	9.9 (6.1-14.1)	>0.05
Priority review (PR)	66	12.8 (9.9-16.1)	90	16.2 (12.1-22.2)	<0.01
Non-designated (ND)	11	19.4 (16.3-24.7)	56	22.5 (17.4-36.8)	>0.05

* Including 9 PR/UNOD dual designations.

Supplemental Table 7. Comparison of NDA approval times between rare disease drugs and non-rare disease drugs

Rare disease drug	Count	NDA approval time (month) Median (IQR)					Comparison *p*-value
		2018-2021	2018	2019	2020	2021	2018-2021 (Yes vs. No)
Yes	21	14.2 (11.6-21.1)	17.9 (12.8-31.1)	14.2 (14.0-20.9)	15.1 (11.9-19.6)	11.8 (9.6-15.4)	>0.05
No	200	15.2 (10.8-21.9)	17.6 (10.4-26.4)	13.7 (11.6-20.4)	16.9 (12.9-21.9)	13.6 (10.0-21.1)	
